# *NRAS* germline variant G138R and multiple rare somatic mutations on *APC* in colorectal cancer patients in Taiwan by next generation sequencing

**DOI:** 10.18632/oncotarget.8885

**Published:** 2016-04-21

**Authors:** Pi-Yueh Chang, Jinn-Shiun Chen, Nai-Chung Chang, Shih-Cheng Chang, Mei-Chia Wang, Shu-Hui Tsai, Ying-Hao Wen, Wen-Sy Tsai, Err-Cheng Chan, Jang-Jih Lu

**Affiliations:** ^1^ Department of Laboratory Medicine, Chang Gung Memorial Hospital at LinKou Taoyuan, Taoyuan, Taiwan; ^2^ Department of Medical Biotechnology and Laboratory Science, Chang Gung University, Taoyuan, Taiwan; ^3^ Department of Colorectal Surgery, Chang Gung Memorial Hospital at Linkou, Taoyuan, Taiwan; ^4^ Graduate Institute of Biomedical Sciences, Chang Gung University, Taoyuan, Taiwan

**Keywords:** next-generation sequencing, colorectal cancer, ampliSeq cancer hotspot panel, NRAS, APC

## Abstract

Colorectal cancer (CRC) arises from mutations in a subset of genes. We investigated the germline and somatic mutation spectrum of patients with CRC in Taiwan by using the AmpliSeq Cancer Hotspot Panel V2. Fifty paired freshly frozen stage 0–IV CRC tumors and adjacent normal tissue were collected. Blood DNA from 20 healthy donors were used for comparison of germline mutations. Variants were identified using an ion-torrent personal genomic machine and subsequently confirmed by Sanger sequencing or pyrosequencing. Five nonsynonymous germline variants on 4 cancer susceptible genes, *CDH1*, *APC*, *MLH1*, and *NRAS*, were observed in 6 patients with CRC (12%). Among them, oncogene *NRAS* G138R variant was identified as having a predicted damaging effect on protein function, which has never been reported by other laboratories. *CDH1* T340A variants were presented in 3 patients. The germline variants in the cancer patients differed completely from those found in asymptomatic controls. Furthermore, a total of 56 COSMIC and 21 novel somatic variants distributed in 20 genes were detected in 44 (88%) of the CRC samples. High inter- and intra-tumor heterogeneity levels were observed. Nine rare variants located in the β-catenin binding region of the *APC* gene were discovered, 7 of which could cause amino acid frameshift and might have a pathogenic effect. In conclusion, panel-based mutation detection by using a high-throughput sequencing platform can elucidate race-dependent cancer genomes. This approach facilitates identifying individuals at high risk and aiding the recognition of novel mutations as targets for drug development.

## INTRODUCTION

Internationally, more than 1 million people are diagnosed for colorectal cancer (CRC) annually, and it is the third leading cause of cancer mortality in Taiwan [1]. CRC arises from a series of sequentially mutated genes that can transform normal epithelial cells into adenoma, eventually becoming adenocarcinoma [2, 3]. The polyp-to-cancer transition takes several years and involves alteration of adenomatous polyposis coli (*APC*), *Kirsten-RAS*(*KRAS*), *TP53*, and other genes that have a role in controlling the cell proliferation process [4, 5]. Except in cases of somatic genomic alteration, CRC can be inherited. The estimated fraction of CRC attributed to inherited predisposition ranges between 10% and 30%. Most cancer-susceptible genes are involved in restraining cell proliferation, DNA repair, and genetic stability [6]. In inherited CRC, defects in the tumor suppressor gene *APC* is a well-known cause of familial adenomatous polyposis (FAP), and hereditary nonpolyposis CRC (HNPCC) is due to germline mutations on DNA mismatch repair (MMR) genes such as *MLH1* and *MSH2* [7, 8].

In the past decade, the success of the Human Genome Project has engendered unprecedented advancements in “precision medicine” [9]. Mutation-derived individual cancer therapy improves patient cure rates. In metastatic CRC, cetuximab or panitumumab, which are monoclonal antibodies and can specifically block epidermal growth factor receptor (EGFR) signal pathways, are effective only in patients with wild-type *KRAS* and *BRAF* [10]. Currently, *KRAS* (Codons 12 and 13) and *BRAF^V600E^* mutation detection are routine molecular companion tests in clinical laboratories before the administration of monoclonal antibody therapy. However, a considerable body of evidence shows that other mutations occurring in genes on EGFR pathways, such as *NRAS* [11] and *KRAS* Codons 61 and 146 [12], are associated with a poor response rate for monoclonal antibody therapy. In addition, the Food and Drug Administration has approved almost 30 types of targeted cancer drugs to specific indications [13], and hundreds of clinical trials are ongoing to develop new drugs targeting certain genes exhibiting specific mutations [14]. Prognosis prediction also relies on specific gene mutation patterns for stratifying patients. For instance, CRC patients with *TP53* mutation, particularly in Codon 175, have a shorter survival period compared with those with wild-type *TP53* [15, 16]. Therefore, developing a high-throughput screening platform that can cover most cancer–related genes is warranted in order to improve the management of patient care.

Recently, targeted next-generation sequencing (NGS) has provided unprecedented potential for detecting underlying changes in the genetic architecture of cancer in a comprehensive and economically feasible manner [17]. The Cancer Genome Atlas study [18] as well as several hospital- and commercial company-based study groups [17, 19, 20] have used NGS platforms to seek specific germline and somatic mutation signatures in CRC, confirming the existence of population-specific mutation patterns [21]. Moreover, race-dependent differences could influence the survival rate of CRC patients under certain conditions [22]. In the present study, we aimed to discover germline and somatic variants in paired tumors and adjacent normal tissues from patients with CRC in Taiwan. Through extensive sequencing on 50 cancer-related genes, a novel germline mutation on oncogene *NRAS,* instead of on well-known cancer-predisposing genes, was observed in our population. Rare somatic variants with frameshift mutations on *APC* and cetuximab resistance mutation on *KRAS* were identified in the tumor tissues. The findings obtained from this type of research can alter the design of gene contents either for screening high-risk individuals with a family history of CRC, or for candidate selection for target therapy.

## RESULTS

### One novel germline variant on *NRAS* and 4 variants on 3 cancer susceptible genes detected in the CRC-adjacent normal tissue samples but not in the asymptomatic controls

To elucidate which genomic alterations were inherited or acquired, paired tumor and adjacent normal tissue samples were collected from 50 patients with CRC. Table 1 lists the clinical features of all enrolled participants. Sixty percent of CRC patients received a diagnosis at early stage (stages 0–II), and the location of tumors was evenly distributed among the right site, left of the large intestine, and the rectum. All patients received surgery to remove tumor lesions. Only one patient with metastatic cancer received target therapy of anti-EGFR monoclonal antibody cetuximab. For an advanced comparison of germline variants between cancer patients and the general population, PBMC DNA samples were collected from 20 young asymptomatic controls with normal CEA and iFOBT lab data and no self-reported cancer history who were recruited for this study.

**Table 1:** Clinical characteristics of the 50 CRC patients and 20 asymptomatic controls

Clinical features		CRC patient	Asymptomatic control
		(n=50)	(n=20)
Sex	Male	27 (54%)	9 (45%)
	Female	23 (46%)	11 (55%)
Age	Median (range)	64 (37-86)	30 (25-48)
CEA	Positive rate	16%	0%
iFOBT	Positive rate	50%	0%
Tumor Stage	0+I	13 (26%)	NA
	II	17 (34%)	NA
	III	19 (38%)	NA
	IV	1 (2%)	NA
Tumor site	Right*	14 (28%)	NA
	Left**	21 (42%)	NA
	Rectum	15 (30%)	NA
Treatment	Surgery	50 (100%)	NA
	Chemotherapy	23 (56%)	NA
	Radiotherapy	3 (6%)	NA
	Target therapy	1 (2%)	NA

* Right site = cancer located in the cecum, ascending colon, transverse colon, and hepatic flexture

** Left site = cancer located in the sigmoid, descending colon, and splenic flexture

NA = not available

Six (12%, 6/50) patients with CRC were identified as carrying germline mutations after being subjected to an analysis pipeline for nonsynonymous variants. Table 2 summarizes the location, annotation, and frequency of the germline and somatic variants in the 6 CRC patients. A total of 5 germline variants were detected on 4 genes, among which a missense variant in the E-cadherin gene (*CDH1)* T340A (COSMIC19821) was present in 3 patients with CRC (Sample ID 1307, 1705, 1738), which represented a germline mutation hotspot in our population (6%, 3/50). The other 2 variants (V1125A and V1352A) on *APC* and one variant (R291Q) on *MLH1* were observed in 3 patients with a family history of CRC. Notably, one novel germline mutation, *NRAS* G138R was observed in Sample ID 1736. In this case, SIFT software selects 122 sequences which are closely related to Homo sapiens *NRAS* and calculate the effect of G138R substitution on NRAS function. It generates a score of 0.01 which is predicted to be deleterious to affect NRAS function. On the other hand, PolyPhen2 software aligns 75 amino acids sequences surrounding the 138^th^ Glycine position from 206 species and found 91.6% of identity in Glycine which means high conservation in this position among species. According to the phylogenetic and structural information of this substitution, this *NRAS* G138R mutation is predicted to be Possibly Damaging with a score of 0.764 (sensitivity: 0.85; specificity: 0.92). This germline variant harbored an oncogene instead of the tumor suppressor gene or DNA repair gene, which are typical in cases of inherited CRC.

**Table 2:** Clinical information of 6 CRC patients and their genomic alterations in paired tumor and adjacent normal tissue samples. Dark blocks highlight the same germline heterozygous variants detected in both compartments

Patient no	Sample ID	Gender	Age at diagnosis	Family history	Adjacent normal				Tumor			
					Gene	Locus	a. a. change	Frequency	Gene	Locus	a. a. change	Frequency
1	1307	Male	64	**Yes**	**CDH1**	**chr16:68846047**	**p.T340A**	**51%**	**CDH1**	**chr16:68846047**	**p.T340A**	**48%**
					**MLH1**	**chr3:37067255**	**p.R291Q**	**50%**	**MLH1**	**chr3:37067255**	**p.R291Q**	**51%**
									**NRAS**	**chr1:115256529**	**p.Q61R**	**67%**
2	1423	Male	78	**Yes**	**APC**	**chr5:112175346**	**p.V1352A**	**50%**	**APC**	**chr5:112175346**	**p.V1352A**	**50%**
									**KRAS**	**chr12:25398284**	**p.G12D**	**39%**
3	1461	Male	47	**Yes**	**APC**	**chr5:112174665**	**p.V1125A**	**49%**	**APC**	**chr5:112174665**	**p.V1125A**	**49%**
				**HNPCC himself**					**ERBB2**	**chr17:37881426**	**p.D873G**	**22%**
									**FGFR1**	**chr8:38285950**	**p.A32D**	**23%**
									**EGFR**	**chr7:55211101**	**p.N115T**	**19%**
									**KDR**	**chr4:55980297**	**p.S265L**	**18%**
									**ATM**	**chr11:108236087**	**p.R3008H**	**20%**
									**PIK3CA**	**chr3:178916876**	**p.R88Q**	**17%**
									**PIK3CA**	**chr3:178916946**	**p.K111N**	**17%**
									**TP53**	**chr7:7578212**	p.R213*	**43%**
									**APC**	**chr5:112174631**	p.R1114*	**38%**
4	1705	Female	73	**Yes**	**CDH1**	**chr6:68846047**	**p.T340A**	**48%**	**CDH1**	**chr16:68846047**	**p.T340A**	**53%**
5	1736	Female	53	NO	**NRAS**	**chr1:115252228**	**p.G138R**	**48%**	**NRAS**	**chr1:115252228**	**p.G138R**	**47%**
									**KRAS**	**chr12:25398284**	**p.G12V**	**35%**
									**APC**	**chr5:112175775**	**p.S1495I**	**73%**
									**APC**	**chr5:112175777**	**p.1496_1498fs**	**73%**
									**APC**	**chr5:112175792**	**p.S1501A**	**73%**
6	1738	Male	84	NO	**CDH1**	**chr6:68846047**	**p.T340A**	**52%**	**CDH1**	**chr6:68846047**	**p.T340A**	**53%**
									**TP53**	**chr5:7578212**	p.R213*	**49%**
									**APC**	**chr7:112175213**	p.1309fs*	**20%**

Four patients with germline mutations had a family history of CRC (Table 2), among whom Sample ID 1461 with *APC* V1125A variant had received a diagnosis of HNPCC. Notably, *APC* is highly associated with FAP and rarely reported in cases of HNPCC. However, this patient developed HNPCC at the age of 47 years, which was younger than most patients with germline mutations in our study.

In general, germline mutations exhibit a mutation frequency of approximately 50% in both normal and tumor tissues. Except for germline variants, 5 of the 6 patients acquired at least one additional somatic mutation in their paired tumor tissues. Notably, Sample ID 1736 gained 4 somatic mutations and Sample ID 1461 gained 9 additional somatic mutations in the tumor samples (except for the inherited *NRAS* G138R and *APC* V1125A variant, respectively). Apart from these 5 patients, only one patient (Sample ID 1705) developed cancer with only one heterozygous germline *CDH1* mutation with the absence of other somatic mutations.

**Figure 1:**
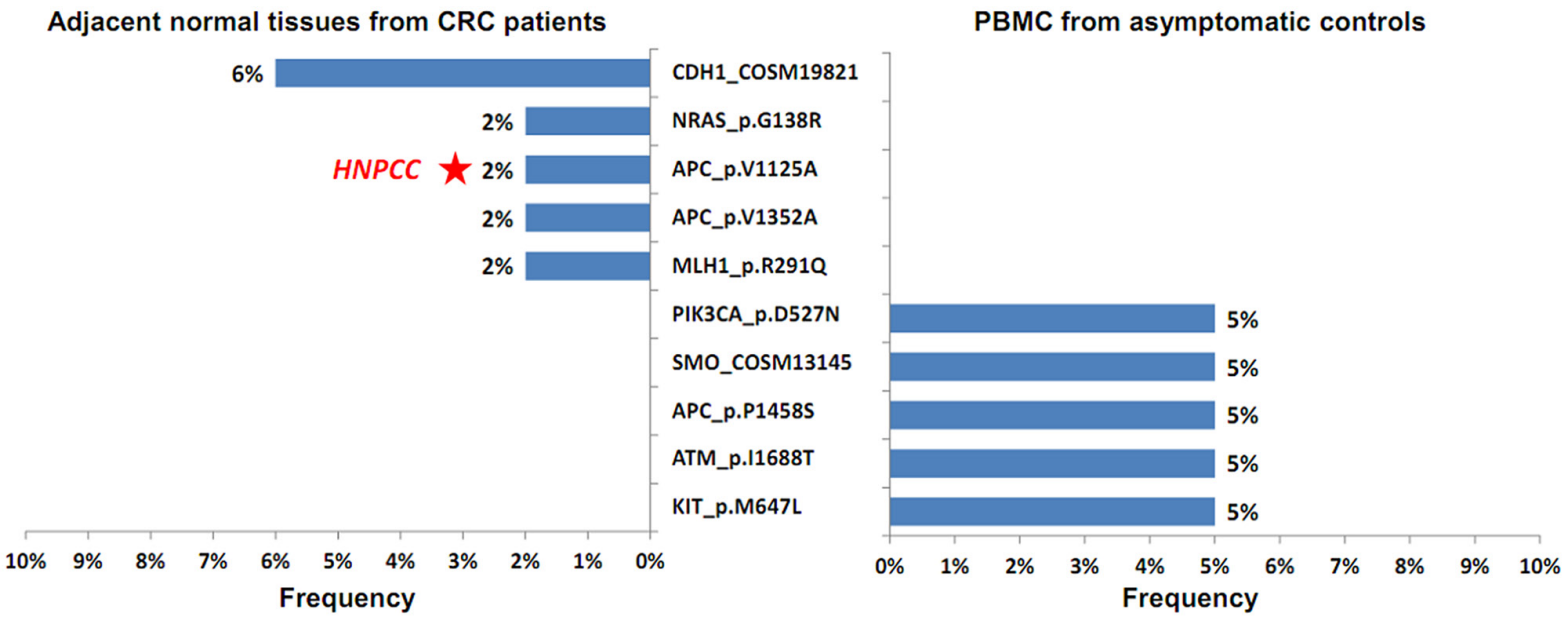
Five unique independent nonsynonymous variants were identified in the 50 normal tissue samples from the CRC patients (left half) and 20 PBMC DNA from the asymptomatic controls (right half) The frequency of each mutant is shown. The red asterisk indicates that the *APC* V1125A germline variant is present in one patient (Sample ID 1461) with diagnosed HNPCC.

To investigate the germline variants in the general population, 20 PBMC DNA samples were collected from the asymptomatic controls and examined. Figure 1 depicts the germline variants obtained from the normal tissues of the CRC patients and controls. Notably, 5 independent variants were observed in each group. Compared with the variants observed in the CRC group, the set of germline alterations in the control group were completely distinct. *SMO* R199W was the only COSMIC variant; the other 4 nonsynonymous variants were predicted as having “tolerated” or “benign” impacts on protein function, and sporadically presented in one individual.

### Twenty-one novel somatic mutations detected in the CRC tumor tissue samples

Initially, 830 variants were identified in the 50 tumor tissue samples. After annotation, only nonsynonymous, frameshift, or stopgain variants affecting the amino acid constitution or function(s) of encoded proteins were filtered. Those variants were further filtered with variants found in adjacent normal tissue samples. Finally, a total of 77 distinct variants on 20 genes were identified and confirmed by Sanger sequencing or pyrosequencing. Among these, 21 (27%) were novel variants not in the COSMIC database. Table 3 ranks the 77 somatic variants by frequency in the 44 CRC patients and provides the serial number of each variant for each gene. According to the variant effect, 56 missense mutations (73%), 13 indel mutations (17%), and 8 nonsense mutations (10%) were identified. Missense mutations of Codons 12 and 13 on *KRAS* (including G12V, G12D, G12S, and G13D) were the most frequently observed variants in the CRC samples (36%, 18/50). Indel and nonsense mutations, which can lead to truncated proteins, were distributed mostly on *TP53* and *APC* genes.

**Table 3:** Seventy-seven somatic variants distributed on 20 genes in CRC tumors. The 21 novel variants not recorded in the COSMIC database are shaded block

Gene	Variant serial no	Coding sequence	Transcript	Variant type	Variant effect	Patient no
TP53	1	c.524G>A	NM_000546	COSM10648_p.R175H	nonsynonymous missense	4
	2	c.637C>T	NM_000546	COSM10654_p.R213*	stop gain	3
	3	c.844C>T	NM_000546	COSM10704_p.R282W	nonsynonymous missense	1
	4	c.818G>A	NM_000546	COSM10660_p.R273H	nonsynonymous missense	1
	5	c.814G>A	NM_000546	COSM10891_p.V272M	nonsynonymous missense	1
	6	c.797delG	NM_000546	COSM44187_p.G266fs	frameshift	1
	7	c.796G>A	NM_000546	COSM10794_p.G266R	nonsynonymous missense	1
	8	c.743G>A	NM_000546	COSM10662_p.R248Q	nonsynonymous missense	1
	9	c.742C>T	NM_000546	COSM10656_p.R248W	nonsynonymous missense	1
	10	c.592G>T	NM_000546	COSM44241_p.E198*	stop gain	1
	11	c.586C>T	NM_000546	COSM10705_p.R196*	stop gain	1
	12	c.536A>G	NM_000546	COSM10889_p.H179R	nonsynonymous missense	1
	13	c.476C>T	NM_000546	COSM11148_p.A159V	nonsynonymous missense	1
	14	c.469G>T	NM_000546	COSM10670_p.V157F	nonsynonymous missense	1
	15	c.455C>T	NM_000546	COSM10790_p.P152L	nonsynonymous missense	1
	16	c.406C>T	NM_000546	COSM11166_p.Q136*	stop gain	1
	17	c.379T>C	NM_000546	COSM44687_p.S127P	nonsynonymous missense	1
	18	c.294_297delTTCC	NM_000546	COSM278467_p.S99fs*23	frameshift +stop gain	1
	19	c.277_278insCCTGGCCCCT	NM_000546	p.L93fs	frameshift	1
KRAS	1	c.35G>T	NM_033360	COSM520_p.G12V	nonsynonymous missense	8
	2	c.38G>A	NM_033360	COSM532_p.G13D	nonsynonymous missense	5
	3	c.35G>A	NM_033360	COSM521_p.G12D	nonsynonymous missense	4
KRAS	4	c.436G>A	NM_033360	COSM19404_p.A146T	nonsynonymous missense	2
	5	c.34G>A	NM_033360	COSM517_p.G12S	nonsynonymous missense	1
	6	c.179G>A	NM_033360	COSM87290_p.G60D	nonsynonymous missense	1
	7	c.183A>C	NM_033360	COSM554_p.Q61H	nonsynonymous missense	1
APC	1	c.2626C>T	NM_000038	COSM18852_p.R876*	stop gain	3
	2	c.3340C>T	NM_000038	COSM13125_p.R1114*	stop gain	2
	3	c.3921_3925delAAAAG	NM_000038	COSM18764_p.E1309fs*4	frameshift+stop gain	1
	4	c.3964G>T	NM_000038	COSM18702_p.E1322*	stop gain	1
	5	c.4330C>T	NM_000038	COSM19021_p.Q1444*	stop gain	1
	6	c.4484G>T	NM_000038	COSM99778_p.S1495I	nonsynonymous missense	1
	7	c.4661dupA	NM_000038	p.E1554fs	frameshift	2
	8	c.3360delA	NM_000038	p.G1120fs	frameshift	1
	9	c.4282delG	NM_000038	p.G1428fs	frameshift	1
	10	c.4285delC	NM_000038	p.Q1429fs	frameshift	1
	11	c.4313_4314insCACCT	NM_000038	p.T1438fs	frameshift	1
	12	c.4348_4357delCGAGAAGTAC	NM_000038	p.1450_1453fs	frameshift	1
	13	c.4475C>T	NM_000038	p.A1492V	nonsynonymous missense	1
	14	c.4486_4493delACTCCAGA	NM_000038	p.1496_1498fs	frameshift	1
	15	c.4501T>G	NM_000038	p.S1501A	nonsynonymous missense	1
PIK3CA	1	c.1633G>A	NM_006218	COSM763_p.E545K	nonsynonymous missense	4
	2	c.263G>A	NM_006218	COSM746_p.R88Q	nonsynonymous missense	2
	3	c.1035T>A	NM_006218	COSM754_p.N345K	nonsynonymous missense	2
	4	c.331A>G	NM_006218	COSM13570_p.K111E	nonsynonymous missense	1
	5	c.333G>T	NM_006218	COSM27505_p.K111N	nonsynonymous missense	1
	6	c.1258T>C	NM_006218	COSM757_p.C420R	nonsynonymous missense	1
PIK3CA	7	c.3073A>G	NM_006218	COSM771_p.T1025A	nonsynonymous missense	1
	8	c.248T>C	NM_006218	p.F83S	nonsynonymous missense	1
	9	c.3073A>C	NM_006218	p.T1025P	nonsynonymous missense	1
SMAD4	1	c.1082G>A	NM_005359	COSM14122_p.R361H	nonsynonymous missense	2
	2	c.353C>T	NM_005359	COSM14215_p.A118V	nonsynonymous missense	1
	3	c.1081C>T	NM_005359	COSM14140_p.R361C	nonsynonymous missense	1
	4	c.1496G>A	NM_005359	COSM14221_p.C499Y	nonsynonymous missense	1
	5	c.344G>A	NM_005359	p.C115Y	nonsynonymous missense	1
	6	c.1586T>C	NM_005359	p.L529S	nonsynonymous missense	1
FBXW7	1	c.1154G>A	NM_018315	COSM117308_p.R385H	nonsynonymous missense	2
	2	c.1273C>T	NM_018315	COSM74637_p.R425C	nonsynonymous missense	1
	3	c.1504T>A	NM_018315	COSM1427667_p.S502T	nonsynonymous missense	1
	4	c.562_563delAT	NM_018315	COSM1052123_p.M188fs*18	frameshift+stop gain	1
NRAS	1	c.182A>G	NM_002524	COSM584_p.Q61R	nonsynonymous missense	2
	2	c.181C>A	NM_002524	COSM580_p.Q61K	nonsynonymous missense	1
BRAF	1	c.1799T>A	NM_004333	COSM476_p.V600E	nonsynonymous missense	1
	2	c.1780G>A	NM_004333	COSM27639_p.D594N	nonsynonymous missense	1
CTNNB1	1	c.121A>G	NM_001904	COSM5664_p.T41A	nonsynonymous missense	1
	2	c.131_133delCTT	NM_001904	COSM33668_p.S45del	frameshift	1
GNAS	1	c.602G>A	NM_001077489	COSM27895_p.R201H	nonsynonymous missense	1
AKT1	1	c.49G>A	NM_001014432	COSM33765_p.E17K	nonsynonymous missense	1
ATM	1	c.9023G>A	NM_000051	COSM21626_p.R3008H	nonsynonymous missense	1
ERBB4	1	c.1825G>A	NM_001042599	COSM131772_p.D609N	nonsynonymous missense	1
FGFR3	1	c.1153T>G	NM_001163213	p.F383V	nonsynonymous missense	1
ERBB2	1	c.2618A>G	NM_004448	p.D873G	nonsynonymous missense	1
FGFR1	1	c.95C>A	NM_023106	p.A32D	nonsynonymous missense	1
PDGFRA	1	c.2470G>A	NM_006206	p.V824I	nonsynonymous missense	1
EGFR	1	c.344A>C	NM_005228	p.N115T	nonsynonymous missense	1
KDR	1	c.794C>T	NM_002253	p.S265L	nonsynonymous missense	1
PTEN	1	c.71A>T	NM_000314	p.D24V	nonsynonymous missense	1

**Figure 2:**
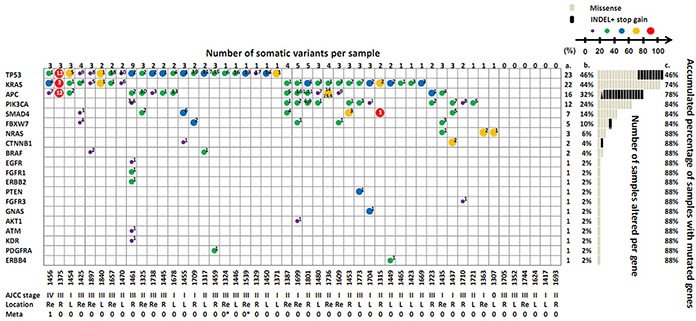
Distribution of 77 somatic variants on 20 genes in the 50 CRC patients The number on the uppermost layer represents the number of somatic variants per sample. The size and color of the circle in the cell represents the individual variant frequency. For interpreting the meaning of each symbol, please refer to the scale bar. The number labeled in the cell indicates the designated variant serial number of each gene, which are listed in Table 3. More than one number in one cell indicates multiple mutations in the same gene. The gray line at the right part of the figure indicates a missense mutation, and the black bar denotes an indel or nonsense mutation. Columns a and b denote the number and percentage of samples altered per gene; Column c denotes the accumulated percentage of samples with mutated genes. The patient information denoted at the bottom of the figure includes the tumor ID, AJCC stage, location, and metastasis condition. “Re” = rectum; “R” = right site; “L” = left site; 1 = metastasis; 0 = no evidence of metastasis; * = follow-up observation of metastasis events 1 year after surgery in 2 stage III patients.

Figure 2 illustrates the distribution of these variants on 20 genes stratified according to each CRC patient. The number of somatic variants in each CRC tumor sample ranged from 0 to 9 with an average of 2.2. The most frequently mutated gene was *TP53* (46%), followed by *KRAS* (44%), *APC* (32%), *PIK3CA* (24%), *SMAD4* (14%), *FBXW7* (10%), and *NRAS* (6%), all of which accounted for 88% of CRC patients. The variant frequency in one tumor can be quantified and marked according to the size and color of the circle in Figure 2. The mutation frequency of each variant in one tumor (in one column) can be compared to clarify the possible clonal expansion history. Most tumors can exert stepwise mutation on various genes. Moreover, we observed multiple mutants in one gene. For example, in the tumor of Sample ID 1736, 3 mutations were observed in the *APC* gene including one 8 nucleotide deletion (Variant Serial Number 14 in Table 3) and 2 missense alterations (Variant Serial Numbers 6 and 15). Other evidence of multiple mutations in one gene can be discovered in the *TP53* and *PIK3CA* genes in Sample IDs 1459, 1461, and 1801.

### Correlation of mutation rate and variant frequency with clinicopathological factors

To investigate the possible correlation between the mutants and disease status, Table 4 summarizes the mutation rate of the top 4 mutated genes and the average variant frequency in the mutated tumors stratified by cancer stage and tumor location. Except for the correlation between the *TP53* mutation rate and the advanced tumor stage (65% vs 33.3%, *P* = *.027*), tumors at a higher stage (stage III and IV) do not exhibit higher mutation rates relative to the total gene mutation rate (85% vs 90% in late stage vs early stage). Patients with tumors in the right site appeared to have a higher *KRAS* mutation frequency (right site vs left site vs rectum, 62% ± 21.6% vs 38% ± 17.6%, 31.9% ± 12.5%, *P* = *.025*), although only a small portion of tumors exhibited *KRAS* mutation in the right site (28.5%). No particular tendency in the mutation pattern at different locations of the large intestine lumen was observed.

**Table 4:** Correlation of the mutation rate and variants frequency of top-4 mutated genes stratified by tumor AJCC stage and tumor location

		AJCC stage			Tumor location			
		Stage 0-II	Stage III-IV	p value	Rectum	Left	Right	p value
		n=30	n=20		n=15	n=21	n=14	
Total genes	Tumor with mutation (%)	90	85	0.594	93.3	85.7	85.7	0.749
	Variants frequency (%)	40.2 ± 18.74	32 ± 20.4	0.046	33 ± 16.8	37.6 ± 19.0	40 ± 22.7	0.226
TP53	Tumor with mutation (%)	**33.3**	**65**	**0.027**	53.3	42.8	42.8	0.793
	Variants frequency (%)	42.7 ± 18.8	41 ± 20.8	0.815	32 ± 12.5	48.4 ± 18.8	43 ± 24.9	0.239
KRAS	Tumor with mutation (%)	46.6	40	0.641	53.3	47.6	28.5	0.369
	Variants frequency (%)	42 ± 15.7	38 ± 25.4	0.692	**31.9 ± 12.5**	**38 ± 17.6**	**62 ± 21.6**	**0.025**
APC	Tumor with mutation (%)	30	40	0.464	53.3	14.2	35.7	0.043
	Variants frequency (%)	41.5 ± 20.1	35.1 ± 25.7	0.638	39.6 ± 24.4	25.6 ± 11.6	45.16 ± 20.8	0.421
PIK3CA	Tumor with mutation (%)	23.3	25	0.892	13.3	19	42.8	0.139
	Variants frequency (%)	31.8 ± 8.67	21.5 ± 5.9	0.031	29.5 ± 7.8	25.7 ± 8.3	27.7 ± 10.9	0.902

### Spatial distribution of the variants in the 4 most frequently mutated genes in the CRC patients and 78% of the novel variants on *APC* can result in frameshifting and early protein termination

**Figure 3:**
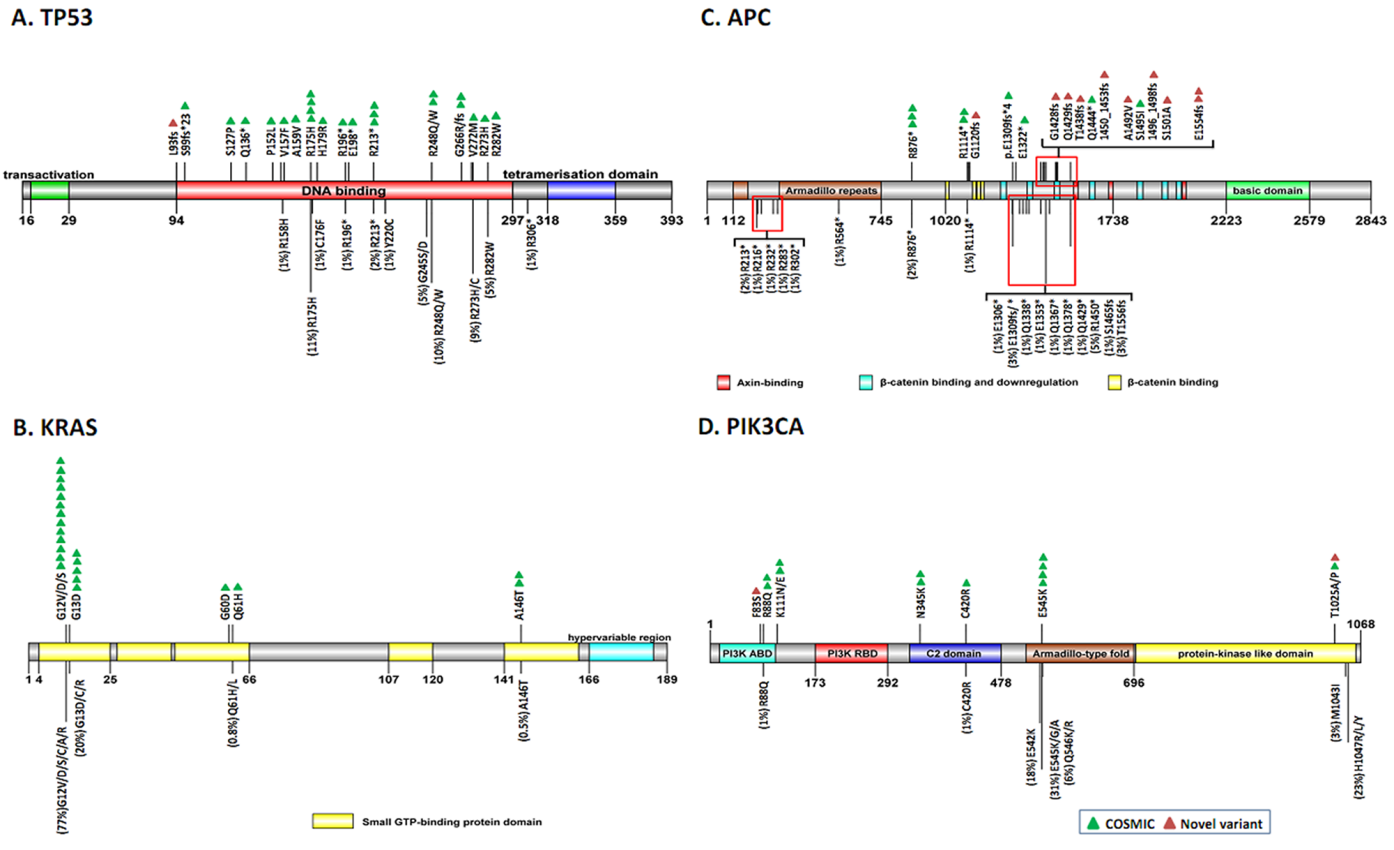
Spatial distribution of somatic variants on the protein function domains of the top-4 mutated genes One triangle represents one variant found in one tumor (green = COSMIC mutation; red = novel variant). Color blocks illustrate the different functional domains of each protein. For comparison, the variants of each gene with a frequency of ≥1% recorded in the COSMIC database are marked with a black line below the domain bar, and the mutation rate is indicated in parentheses and indicated by the length of the line. **A.** *TP53*; **B.** *KRAS*; **C.** *APC*; **D.** *PIK3CA.*

Figure 3 illustrates the spatial distribution of the variants according to protein function domain in the 4 most frequently mutated genes in the CRC patients. Reported mutations and their frequency extracted from the COSMIC database are also shown under the protein domain bar for comparison. R175H on *TP53*, mutation of Codons 12 and 13 on *KRAS*, and E545K on *PIK3CA* were the highest frequency mutations in our population (detected in at least 4 patients, marked with a green triangle in Figure 3). All of these mutations also contributed to the most frequently reported mutations in the COSMIC database (11%, 97%, and 31% in each gene, indicated by the line length in the figure). Otherwise, the remaining mutations were distributed widely among the cancer-related genes with no obvious hotspot. However, all mutations on the *TP53* gene were located in the DNA-binding domain, and the variants on the *APC* gene were distributed in the β-catenin-binding domain. Both domains are central parts of the TP53 and APC proteins and govern the tumor suppression function by binding the downstream ligands. Notably, we unveiled 21 novel variants in 11 genes, 9 of which were observed in the *APC* gene (marked by a red triangle in Figure 3) and were located in the mutation cluster region (MCR). Furthermore, 78% (7/9) of these novel variants on the *APC* gene can result in a frameshift and early protein termination and may lead to deleterious consequences.

## DISCUSSION

In this study, we established an analysis pipeline for a well-designed cancer hotspot panel on an IT-PGM platform and comprehensively investigated the germline and somatic variants in CRC patients in a Taiwanese population. The results reveal the existence of 5 germline variants specifically in cancer patients but not in the general population. Among the 5 germline mutants, *NRAS* G138R is a novel mutation that has never been reported, and *CDH1* T340A was the most frequently occurring mutation in the CRC group (Figure 1). In addition, 21 novel somatic variants were identified in the tumor samples, among which 7 mutations were frameshift alterations located in the central part of the *APC* gene, which may alter the function of the APC protein (Table 3).

In studying the germline mutations, we unexpectedly determined a novel *NRAS* G138R in one CRC patient. In well-investigated inherited cancers, such as breast cancer and colon cancer, germline mutations frequently occur in tumor suppressor genes (eg, *APC*, *STK11*, and *PTEN*) and DNA repair genes (eg, *MLH1*, *MSH2*, *BRCA1*, and *BRCA2*) [3, 27]. Inherited mutations in oncogenes, which can be predisposed to cancer, have only been reported on missense mutations of the *RET* proto-oncogene for multiple endocrine neoplasia type 2A cancer syndrome [28]. For our understanding, this is the first study to report the finding of germline *NRAS* mutations in CRC patients. *NRAS* belongs to *the RAS* oncogene family (*KRAS*, *HRAS*, and *NRAS*) and Ras protein functions as a GTPase, which can conduct signal transduction from the outside of the cell to the nucleus. Previous literatures have reported that the 138^th^ residue is located in the allosteric lobe of Ras protein (residues 87-171) and is responsible for the interaction with GTPase-activating proteins (GAPs) [29]. GAPs interact with Ras-GTP by insertion of the arginine finger into the active site to switch “on” the GTP hydrolysis. Based on this model, the Glycine to Arginine substitution found in this study would possibly enhance downstream signaling. Moreover, the mutation was predicted to have a damaging effect on Ras protein function, as determined by SIFT and Polyphen2 software. However, in vitro function test of this mutant is needed to prove the cancer-prone property.

The remaining germline mutations were located in 3 cancer-susceptible genes: *CDH1*, *APC*, and *MLH1*. Among these, *CDH1* mutation has been recognized as a risk for early onset diffuse gastric cancer in Western countries [30]. However, *CDH1* T340A was reported to have a high association only with inherited and sporadic colon cancer in a Korean population [31]. As many as 3 patients in our CRC group also carried this specific heterozygous germline mutation (Table 2), indicating the existence of an Asian-specific genome structure. Another finding of interest is the role of *APC* in inherited CRC. Although *APC* is a known causal gene for FAP with high penetrance, the specific V1125A substitution and another V1352A mutation observed in our study was previously reported in a cohort study comprising 480 non-FAP patients [32]. Compared with CRC, most *de novo* germline mutations in the general population are not related to cancer development and have been designated to benign alteration by protein function prediction software. Recently, a commercial laboratory in the United States reported descriptive findings from screening inherited CRC from 586 patients by using the ColoNext™ NGS panel, which recruits 14 genes [20]. That study showed that 71% of patients with pathogenic mutations on CRC-susceptible genes met the syndrome-based testing criteria. However, their panel did not include *NRAS* and no Asian patients had positive findings. In summary, the result of this study highlight the importance of establishing a population-specific screening panel to maximize the detection rate of germline mutation for cancer prevention.

Regarding somatic mutation, an average of 2.2 variants can be identified in each tumor. However, 12 patients harbored only one aberrant mutation in the *TP53*, *KRAS*, *PIK3CA*, or *NRAS* gene in their tumor tissues (Figure 2), emphasizing the dominant role of these genes in carcinogenesis. Among all the variants, 21 somatic variants (27%) were unique to Taiwanese CRC patients (Table 3). Furthermore, 9 of these 21 variants were located in the *APC* gene, 7 of which belong to the indel-type, which can cause truncation of the encoded APC protein. The full length of *APC* acts as a tumor suppressor protein, which can disconnect the Wnt signal pathway by forming a multiprotein complex with Axin and β-catenin and promoting the phosphorylation and subsequent degradation of β-catenin [33]. More than 90% of the reported *APC* mutations found in sporadic and inherited CRC are nonsense or frameshift mutations located in the β-catenin-binding region (MCR: Codons 1267-1529), and the resulting shorter protein may lose its ability to bind to β-catenin, thereby activating cell proliferation and migration [34]. In the present study, 9 novel mutations on the *APC* gene were distributed from Codons 1120 to 1554 and might contribute to carcinogenesis. Moreover, a small-molecule compound was identified recently as selectively poisoning cancer cell lines with truncated *APC* [35], which might benefit the patients with *APC* novel frameshift mutations in our population. Other non-COSMIC mutations were involved in EGFR-RAS, PI3K, and P53 signaling pathways.

The variant profile in each patient provided an opportunity to inspect the inter- and intra-heterogeneous nature of CRC tumors [36] by examining 77 variant types distributed in 44 patients with CRC. We observed only 2 patients (Sample IDs 1423 and 1669) had the same mutation profile with *KRAS* G12D alteration. The remaining 42 patients had unique mutation signatures (Figure 2). This high inter-tumor heterogeneity may be induced by the stochastic nature of genome damage during passage through differences in tumor-initiating insults, immune surveillance, and factors influencing cancer progression [37]. The heterogeneity explains the loose correlation between genotype and phenotype (Table 4). However, concordance to our finding, higher levels of *TP53* mutant DNA [38] or mutant p53 protein [39] can be found in late AJCC stage of CRC tumors. One large cohort study which recruited 1110 Chinese CRC patients revealed that mutant *KRAS* and *BRAF* were associated with right-sited tumors [40] and it correlated with the poor response to epidermal growth factor receptor (EGFR) inhibition with cetuximab [41].

On the other aspect, intra-tumor heterogeneity can be demonstrated by observing the highly extreme variant frequency in one tumor and the discrepant finding of common serial mutation order from *APC* to *TP53* which advised by Bert Vogelstein in 1988 [3]. According to the theory, the earliest event in the colorectal cancer involves the mutation of *APC* gene. Acquiring and accumulating more somatic mutations on specific genes is essential for malignant transformation (Supplementary Figure S1A). However, the variant frequency on *APC* gene in each tumor is not always the largest one in our study. This phenomenon provides a clue that the *APC* may not be the necessary driver gene. Another subclones which carry the essential mutations gain growth advantage as tumor progression (Supplementary Figure S1B). Both conditions can occur in one tumor and cause the intra-tumor heterogeneity. The nature of intra-tumor heterogeneity may hinder the correct mutation profile detection and the subsequent choice for personalized target therapy. Consequently, it could be at the risk of introducing the propagation of minor clones in the original tumor after incomplete therapy. However, the impact and clinical correlation of intra-tumor heterogeneity needs larger sample cohort and long-term follow-up study.

The only one stage IV patient (Sample ID 1456) who received palliative chemotherapy and cetuximab in this study showed progression of bone metastasis 1 year later. After examination of the mutation profile of this patient by using NGS Cancer Panel, one rare *KRAS* mutation was observed at A146T. This mutation accounts for only 0.5% of all *KRAS* mutations in the COSMIC database. However, a recent study indicated that this mutation reduces the sensitivity to anti-EGFR antibody therapy [12]. The examination of other drug-actionable targets through NGS cancer panel shows promise for cancer patients to receive the newest therapy, even for drugs undergoing clinical trials. Park applied NGS to 2221 clinical cases and observed clinically actionable alterations in 76% of tumors, which is 3-fold the number of actionable alterations detected by conventional diagnostic tests [42]. These findings strengthen the necessity of implementing an NGS cancer panel in clinical settings instead of conventional PCR strategies for detecting hotspots.

Collectively, the unexpected germline oncogene mutation and high frequency of novel variants observed in our cancer group may have an ethnic impact [43]. The clinical implementation of the NGS cancer panel, either in germline or somatic genome detection, is currently under development in our laboratory with the aim of improving the screening rate of cancer for high-risk individuals with a family history of CRC and providing more actionable information for physicians to improve medical care for CRC patients.

## MATERIALS AND METHODS

### Study patients

#### CRC patients

Fifty patients with untreated CRC diagnosed in 2013 at Chang Gung Memorial Hospital in Taiwan were enrolled in this study. Cancer was staged according to the 2009 American Joint Committee on Cancer staging criteria (7th edition) [23]. Clinicopathological factors, including age, sex, plasma carcinoembryonic antigen (CEA) data, immuno-occult blood test (iFOBT) data, and the anatomic subsite of tumors in the intestine lumen, were recorded at enrollment. Tumor location was classified into 3 parts: right site (tumors at the cecum, ascending colon, hepatic flexure, and transverse colon); left site (tumors at the splenic flexure, descending colon, and sigmoid colon); and the rectum.

#### Asymptomatic controls

For the control group, we recruited 20 volunteers from staffs at the Department of Laboratory Medicine at Linkou Chang Gung Memorial Hospital. All controls had no family history of CRC and had negative serum CEA and iFOBT results. All patients and healthy individuals were provided with a form of written informed consent, and the study was approved by the Institutional Review Board of Chang Gung Memorial Hospital (101-4609A3).

### Sample preparation and routine laboratory test

Fresh tumors and adjacent normal tissue samples (at least 5-cm from the tumor site) were collected on the day of operation from 50 patients with CRC. For the control group, the EDTA blood samples were collected from 20 asymptomatic controls. Genomic DNAs were extracted using a DNeasy Blood and Tissue Kit (Qiagen, Valencia, CA, USA) and stored at −80°C until use. CEA and iFOBT were respectively determined using an ADVIA Centaur® Analyzer (WI, USA) with a cutoff of 5 ng/mL and OC-Sensor Diana Latex Reagent (Eiken Chemical, Tokyo, Japan) with a cutoff of 100 ng/mL.

### Ion-torrent personal genome machine (IT-PGM) sequencing

AmpliSeq Cancer Hotspot Panel Version 2 (Life Technologies, CA, USA) specifically targets 50 cancer-related genes, most of which are tumor suppressor genes and oncogenes, and harbors 2855 COSMIC (Catalogue of somatic mutations in cancer) [24] hotspots (for detailed information, see Supplementary Table S1). The AmpliSeq Library was prepared according to the manufacturer's Ion AmpliSeq Library Kit protocol. In brief, 10 ng of genomic DNA was extracted from the samples and PCR was conducted to amplify 207 amplicons, with sizes ranging from 49 to 140 bp, in one primer pool. After AMPure bead purification, barcoded adapter-ligated products were nick-translated, and the resulting library concentration was determined using an Agilent 2100 bioanalyzer and adjusted to 10 pmole. Emulsion PCR and enrichment were performed on an Ion One Touch System by using the Ion OneTouch™ 200 Template Kit Version 2.0 (Life Technologies) according to the manufacturer's instructions. The samples were then sequenced using the IT-PGM 200 Sequencing Kit Version 2.0 protocol. To obtain an average depth of 1500 for each amplicon, 6 samples were pooled on one 316 chip, and 12 samples were pooled on one 318 chip.

### Bioinformatics analysis

Base sequences were processed initially by using IT-PGM pipeline software (Torrent Suite Version 4.2), and the sequences were aligned to human genome build 19 reference genome (hg19). Identification of variants was facilitated by using the IT Variant Caller software plugin (Life Technologies), and advanced annotation was performed by uploading an exported VCF file from Variant Caller to Vanno (developed by the Department of Bioinformatics at Chang Gung University) [25]. Initially, variants were selected by mutation type if they belonged to nonsynonymous or frameshift or stopgain at the exonic region. Variant frequencies >3% in the dbSNP-Asian database were further filtered. To enhance the reliability of these variants, only those mutations with a frequency of >5% and variant coverage of >30 were considered candidate variants for further analysis. Integrative Genomics Viewer was employed to visualize the variants by confirming the presence of possible strand biases and alignment errors. Nomenclature of novel variants followed the rules from Human Genome Variation Society (http://www.hgvs.org/mutnomen/). Variants with amino acid changes were further examined for whether the changes were potentially damaging alterations by using Sorting Tolerant From Intolerant (SIFT) and Polymorphism Phenotyping v2 (PolyPhen2) software, which can predict the possible impact of an amino acid substitution on the structure and function of a protein [26]. SIFT calculates conservation value and scales probability for each position. The SIFT score ranges from 0.0 (deleterious) to 1.0 (tolerated). The PolyPhen-2 score ranges from 0.0 (tolerated) to 1.0 (deleterious).

### Variant confirmation

Mutations that met the filtering criteria were further confirmed by Sanger sequencing when the variant frequency was above 20%, or by pyrosequencing when the variant frequency was 5%–20%.

### Statistical analysis

Descriptive statistics were summarized in percentages, ranges, means, and standard deviations. Between-group comparisons were conducted using the Student *t* test, one-way ANOVA, and chi-square test for each marker. A *P* value less than 0.05 (2-tailed) was considered statistically significant. All statistical tests were conducted using PASW Statistics 18. Protein domain structure and distribution of variants on specific proteins were plotted using DOG Version 1.0 (http://dog.biocuckoo.org).

## SUPPLEMENTARY FIGURE AND TABLE


