# Supplementary material for: NRAS germline variant G138R and multiple rare somatic mutations on APC in colorectal cancer patients in Taiwan by next generation sequencing
Source: Oncotarget. 2016 Apr 21;7(25):37566–80. doi: 10.18632/oncotarget.8885 (PMC5122332; doi:10.18632/oncotarget.8885)
Supplement: Supplementary file 1 [file oncotarget-07-37566-s001.pdf]

NRAS germline variant G138R and multiple rare somatic mutations on *APC* in colorectal cancer patients in Taiwan by next generation sequencing

SUPPLEMENTARY FIGURE AND TABLE

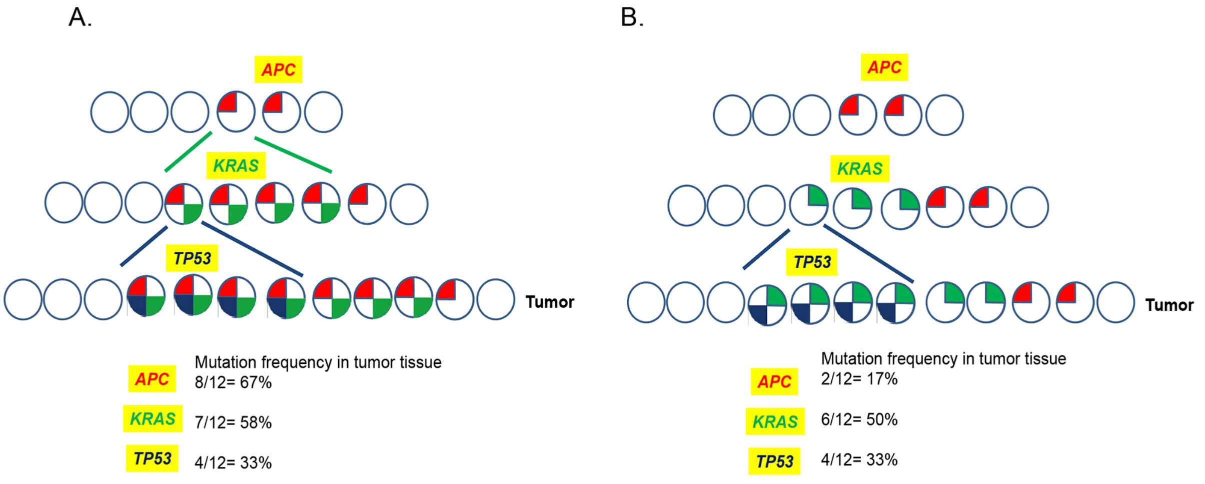

Supplementary Figure S1: Illustration of intra-tumor heterogeneity by two possible mutation pathways. A. Clone successions from one cell with initial mutation in *APC* gene (red) to final malignant clones with further obtained second and third mutation in *KRAS* and *TP53* (green and blue). The sum of mutation percentage of three serially mutated genes in tumor tissue are 67%, 58% and 33% (tentative cell number). **B.** Cells with *KRAS* mutation only may have more proliferation advantage over the cells with *APC* mutation or cells lacking any mutation. Subsequent mutation may occur in clones with *KRAS* mutation only. The final genotype will be *KRAS* dominant instead of *APC*.

Supplementary Table S1: Distribution of 2855 hotspots of Cancer Hotspot Panel v2 in 50 cancer associated genes. Corresponded drugs of each gene for target therapy and FDA-approved indication to specific cancer list at the right two columns

No	Gene	COSMIC hot spot number in panel (n=2855)	Oncogene or tumor suppressor gene	Drug for target therapy	FDA-approved indication(s)
1	TP53	1150	Tumor Suppressors		
2	APC	164	Tumor Suppressors		
3	PTEN	159	Tumor Suppressors		
4	KIT	139	Oncogene	Axitinib (Inlyta)/Pazopanib (Votrient)Cabozantinib (Cometriq)/Sorafenib (Nexavar)Imatinib (Gleevec)/ Regorafenib (Stivarga)	Renal cell carcinomaMedullary thyroid cancerGI stromal tumor (KIT+)
5	VHL	124	Tumor Suppressors		
6	EGFR	123	Oncogene	Gefitinib (Iressa)/Afatinib (Gilotrif)Cetuximab (Erbitux)/Panitumumab (Vectibix)Erlotinib (Tarceva) Vandetanib (Caprelsa)	NSCLC (with mutations)Colorectal cancer (KRAS/BRAF wild type)NSCLC/ Pancreatic cancerMedullary thyroid cancer

(*Continued*)

No	Gene	COSMIC hot spot number in panel (n=2855)	Oncogene or tumor suppressor gene	Drug for target therapy	FDA-approved indication(s)
7	CDKN2A	108			
8	PIK3CA	97	Oncogene	PI3 kinase/mTOR inhibitors (under trial)	
9	BRAF	77	Oncogene	Dabrafenib (Tafinlar)/ Vemurafenib (Zelboraf) Resistance to Cetuximab/ PanitumumabBRAF inhibitor (under trial)	Melanoma (with BRAF V600 mutation)
10	CTNNB1	73	Oncogene		
11	KRAS	63	Oncogene	Resistance to Cetuximab/ PanitumumabMEK inhibitor (under trial)	
12	NRAS	35	Oncogene		
13	SMAD4	31	Tumor Suppressors		
14	FLT3	30	Tumor Suppressors	Cabozantinib (Cometriq) Ponatinib (Iclusig)	Medullary thyroid cancerCML /ALL (with Philadelphia chromosome)
15	NPM1	28	Tumor Suppressors		
16	PTPN11	28	Oncogene		
17	HRAS	27	Oncogene		
18	PDGFRA	26		Imatinib (Gleevec)Pazopanib (Votrient)	Dermatofibrosarcoma protuberans Renal cell carcinoma
19	FBXW7	25	Tumor Suppressors	potential resistance to tubulinsmTOR inhibitor (under trial)	
20	ATM	24	Tumor Suppressors	PARP inhibitors (under trial)	
21	STK11	23	Tumor Suppressors		
22	NOTCH1	20	Tumor Suppressors		
23	ABL1	19	Oncogene	Bosutinib (Bosulif)/Dasatinib (Sprycel)Imatinib (Gleevec)/ Nilotinib (Tasigna)Ponatinib (Iclusig)	CML /ALL (with Philadelphia chromosome)
24	ERBB2	19	Oncogene	Trastuzumab (Kadcyla)/ Pertuzumab (Perjeta)/ Trastuzumab (Herceptin) Lapatinib (Tykerb)	Breast cancer (HER2+)
25	RB1	18	Tumor Suppressors		
26	MET	18	Oncogene	Cabozantinib (Cometriq) Crizotinib (Xalkori)	Medullary thyroid cancerNSCLC

(*Continued*)

No	Gene	COSMIC hot spot number in panel (n=2855)	Oncogene or tumor suppressor gene	Drug for target therapy	FDA-approved indication(s)
27	RET	17	Oncogene	Cabozantinib (Cometriq)/ Vandetanib (Caprelsa) Regorafenib (Stivarga)	Medullary thyroid cancerColorectal cancer
28	FGFR3	17			
29	IDH1	15	Oncogene		
30	ERBB4	13	Oncogene		
31	GNAS	12		MEK or ERK inhibitors (under trial)	
32	IDH2	12			
33	EZH2	11			
34	KDR	11			
35	SMARCB1	11	Tumor Suppressors		
36	HNF1A	10			
37	MPL	10	Oncogene		
38	ALK	8		Ceritinib (Zykadia)Crizotinib (Xalkori)	NSCLC (with ALK fusion)
39	CSF1R	8			
40	FGFR2	8	Oncogene		
41	CDH1	7	Tumor Suppressors		
42	AKT1	6	Oncogene		
43	GNAQ	6			
44	JAK3	6		Tofacitinib (Xeljanz)	Rheumatoid arthritis
45	GNA11	5			
46	JAK2	5	Tumor Suppressors	Ruxolitinib (Jakafi)	Myelofibrosis
47	SMO	5	Oncogene		
48	FGFR1	2	Oncogene	Ponatinib (Iclusig)	CML /ALL (with Philadelphia chromosome)
49	MLH1	1	Tumor Suppressors		
50	SRC	1			
